# Towards achieving semantic interoperability of clinical study data with FHIR

**DOI:** 10.1186/s13326-017-0148-7

**Published:** 2017-09-19

**Authors:** Hugo Leroux, Alejandro Metke-Jimenez, Michael J. Lawley

**Affiliations:** The Australian E-Health Research Centre, CSIRO Health and Biosecurity, Level 5, Health Sciences Building 901/16, Royal Brisbane and Women’s Hospital, Herston, 4029 Queensland Australia

**Keywords:** FHIR, CDISC ODM, Interoperability, Clinical research data, Longitudinal clinical study

## Abstract

**Background:**

Observational clinical studies play a pivotal role in advancing medical knowledge and patient healthcare. To lessen the prohibitive costs of conducting these studies and support evidence-based medicine, results emanating from these studies need to be shared and compared to one another. Current approaches for clinical study management have limitations that prohibit the effective sharing of clinical research data.

**Methods:**

The objective of this paper is to present a proposal for a clinical study architecture to not only facilitate the communication of clinical study data but also its context so that the data that is being communicated can be *unambiguously* understood at the receiving end. Our approach is two-fold. First we outline our methodology to map clinical data from Clinical Data Interchange Standards Consortium Operational Data Model (ODM) to the Fast Healthcare Interoperable Resource (FHIR) and outline the strengths and weaknesses of this approach. Next, we propose two FHIR-based models, to capture the metadata and data from the clinical study, that not only facilitate the *syntactic* but also *semantic* interoperability of clinical study data.

**Conclusions:**

This work shows that our proposed FHIR resources provide a good fit to semantically enrich the ODM data. By exploiting the rich information model in FHIR, we can organise clinical data in a manner that preserves its organisation but captures its context. Our implementations demonstrate that FHIR can natively manage clinical data. Furthermore, by providing links at several levels, it improves the traversal and querying of the data. The intended benefits of this approach is more efficient and effective data exchange that ultimately will allow clinicians to switch their focus back to decision-making and evidence-based medicines.

**Electronic supplementary material:**

The online version of this article (doi:10.1186/s13326-017-0148-7) contains supplementary material, which is available to authorized users.

## Background

Clinical research plays a vital role in advancing medical knowledge and improving clinical outcome. It is becoming increasingly clear that results from clinical studies need to be shared and compared to one another in order to support efficient evidence-based medicine [1] and reduce the costs of conducting these studies. By the same token, Hsu et al. [2] argue that to fulfil the goals of precision medicine requires the mining and aggregation of clinical data from multiple sources and entails novel approaches to obtaining contextual observations. Hume et al. [3] state that: “*clinical research can no longer be considered an isolated venture and is increasingly conducted in network structures where seamless data exchange is critical to operational efficiency and effectiveness*”. The challenge when comparing results from different data sets is to ensure that we are comparing corresponding data sets.

The Operational Data Model (ODM) [4] is an XML^1^-based standard from the Clinical Data Interchange Standards Consortium (CDISC) that was originally developed to facilitate the exchange, archival and audit trail requirements of clinical information but whose use has been extended to cover cases not initially anticipated [3], such as integrating health records within clinical research systems. The Federal Drug Administration has mandated the use of the CDISC standards for the electronic capture and reporting of clinical study data [5]. ODM is particularly well-suited for a data capture context [3, 6–11]. It is a mature data interchange standard that has proven useful for exchanging both document and message formats [3]. Its strength is in its relative simplicity, adaptability through the use of extensions [3] and in its ability to support the creation of a broad range of customisable Clinical Report Forms (CRFs) [3, 12].

ODM, however, lacks a rich-enough information model to capture the innate contextual information of the clinical study data [7, 13]. Its relative simplicity, has impacted on its ability to advance all aspects of interoperability, limiting its support for data mapping, data types, terminology and semantic representation [3]. In spite of its efficacy as a data interchange, ODM has some shortcomings in the mapping of semantically identical data elements due to lack of support for semantics associated with the data elements [3, 11].

ODM can be considered to represent *syntactic interoperability* (as defined by [14]) of clinical data as it provides a vehicle for clinical data to be shared using an XML-based model. However, our aim is to achieve semantic interoperability. *Semantic interoperability* is the ability, for health information systems, to exchange information and automatically interpret the information exchanged meaningfully and accurately in order to produce useful results as defined by the end users of both systems [14, 15]. Extensions to ODM, such as the Clinical Data Acquisition Standards Harmonization (CDASH) [16] and the Biomedical Research Integrated Domain Group (BRIDG) [17] provide a reference model, although as stated by [18]: “*studies that use CDASH CRFs achieve semantic alignment through a shared data standard, rather than through specific semantics*”. Furthermore, there is no requirement for the CDASH model to be used within ODM [7]. Moreover, uptake of CDASH and BRIDG to provide data semantics has been limited [3]. As a result, ODM is ill-suited for advancing the *semantic interoperability* solution that is required to achieve cross-study exploration of the clinical studies as there is the potential for the data to be interpreted in a way that was not originally intended by the study initiators.

The ability to achieve cross-study analysis also necessitates clinical studies to adopt a more streamlined data structure [7]. However, the monolithic nature of the ODM data model favours a one-dimensional traversal of the clinical data along its hierarchy of Study-Subject-StudyEvent-Form-ItemGroup-Item. More effective exploration and querying of the clinical data, especially when dealing with longitudinal studies, requires more direct access to the data, particularly at the Study Event, Subject and Item levels [6, 7, 19].

The Fast Healthcare Interoperable Resources [20] (FHIR) framework, a HL7^2^ standard that has been swiftly adopted by the health-care community [21–23], looks the likely candidate for overcoming this challenge. It is geared towards communication of clinical data using HL7 messaging protocols but is also supported by a rich information model to achieve semantic interoperability of clinical data. This makes FHIR the natural match to complement the ODM standard [8] as ODM shares several design principles, such as making use of extensions for edge cases and human readability, with FHIR [3]. Furthermore, FHIR has the potential to incorporate existing electronic health record (EHR) data to augment the findings of *retrospective observational* studies. As intimated by Kubick [24], “*FHIR can make it possible to reach inside of EHRs not just to capture data, but to monitor protocol progress, provide safety alerts, and allow much greater visibility into trial conduct and can lead to dramatic improvements in study efficiency and drug safety*”.

This research builds upon the approach [8] to integrate clinical data extracted in CDISC ODM format into several FHIR resources with a view to achieving semantic interoperability of clinical study data. In the next section, we outline the approach taken to map the ODM-based data and metadata onto eight FHIR resources. In particular, we outline the suitability of the FHIR resources in supporting the ODM model and on all the assumptions made to reintroduce the contextual information to the data. We then critique this approach. Consequently, we propose the FHIR ClinicalStudyPlan resource to capture the clinical study metadata, including the potential to encode the study protocol as part of the model. This is followed by a description of the FHIR ClinicalStudyData resource that describes the clinical study data. Finally, it leads into a discussion on the design principles of the two proposed FHIR resources and on their suitability for representing clinical study data.

## Integrating ODM with FHIR

**Fig. 1:**
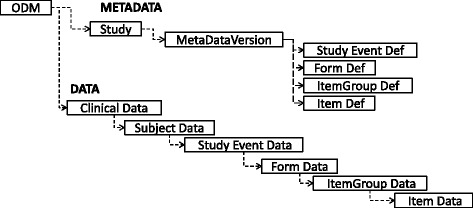
The ODM data model. Illustrates the logical organisation of the ODM model into the *data* and *metadata* hierarchies

**Fig. 2:**
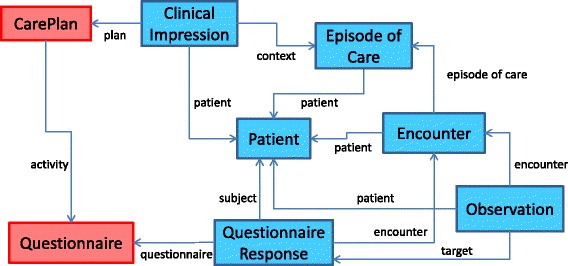
The FHIR data model. Depicts the *metadata* (red) and *data* (blue) FHIR resources and their links that comprise the data model to transform the clinical data from ODM to FHIR. The CarePlan and Questionnaire resources are used to capture the *metadata* for the study. A Patient resource is used to represent the study participant while the clinical data for this participant is contained within a ClinicalImpression resource. The study events are captured within the EpisodeOfCare resource and the Encounter resource represents one atomic event. The QuestionnaireResponse resource captures the form responses and the Observation resource illustrates those responses that are analogous to a patient’s observations. The QuestionnaireResponse resource is linked back to the Questionnaire resource

This section outlines the approach described in [8] to integrate the ODM data model to a selection of eight FHIR^3^ resources to capture both the data and metadata properties of the ODM data model. The CDISC ODM data model [4] consists of two main hierarchies: a Clinical Data and a Metadata hierarchy, as depicted in Fig. 1, that are referenced using the same object identifier (OID). These two parallel hierarchies ensure that the clinical study follows a predetermined structure of subject, event, form, item group and item. Figure 2 outlines the FHIR resources chosen to model the ODM data. The entities in red (CarePlan and Questionnaire) denote metadata concepts. The remaining entities, in blue, model the clinical data at various levels of the ODM hierarchy. Solid lines are used to denote the links between the entities.

**Fig. 3:**
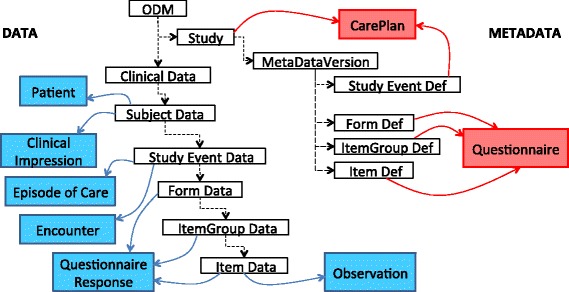
Mapping the ODM data model to the FHIR resources. Illustrates how the CDISC ODM model (depicted by unshaded rectangles) is overlaid with the FHIR resources. The Metadata section, depicted on the right of the model with *red* rectangles to represent the FHIR resources, is mapped to the CarePlan resource at the Study and Study Event level, and to the Questionnaire resource to represent the form and its composition. The Data section is depicted on the left of the model with the FHIR resources depicted as *blue* rectangles. The Patient resource represents the study participant. The ClinicalImpression resource captures the clinical data for this participant and they are both linked to the ODM model at the Subject Data level. As both the EpisodeOfCare and Encounter resource correspond to study events, they are mapped at the StudyEventData level. The QuestionnaireResponse resource captures the form responses and is linked to the form data and its composition. Finally, the Observation resource is used to capture those responses that are more analogous to a patient’s observations

The approach taken to map the ODM data into FHIR resources is a *semi-automatic* process. As the ODM data model does not natively provide any mechanism to capture the contextual information relating to the study, the data semantics needs to be re-introduced during this process. This can only be achieved if the person doing the mapping has access to all the conceptual information defining the study. Figure 3 illustrates how the hierarchical ODM model has been mapped to the FHIR resources.

### Study

A study defines static information about the structure of an individual study. We choose to model the Study component from ODM using the CarePlan resource because we want to model the activities planned for the patient during the study in the context of the study protocol. CarePlan provides a link to the study coordinator through the participant attribute and study protocol through the support attribute. Furthermore, the CarePlan resource offers a number of attributes, such as context, category and description that can provide additional context to the care plan.

### Subject

While the Subject represents a critical element of the study, its role is quite subdued in ODM. In particular, the specification provides no functionality to record the subject’s attributes such as gender or date of birth, recommending that these be modelled as clinical data within the forms. The logical mapping for the Subject in FHIR is the Patient resource. Relevant contextual information, such as the patient’s gender, date of birth and care provider, can be encapsulated within the resource. The clinical data for each subject is contained within a ClinicalImpression resource that is linked to the Patient resource. The care plan is linked to this resource using the plan attribute. The ClinicalImpression permits very pertinent information to be associated to the patient’s data through the use of the trigger, investigations and summary attributes.

### Study event

A study event comprises a StudyEventDef and a StudyEventData component that are referenced using a common OID. The StudyEventDef manages the set of forms to be completed at this phase of the study and represents an activity within the CarePlan resource. StudyEventDef entities define *scheduled* and *unscheduled* events and these are defined within the detail.scheduled attribute of the activity. The StudyEventData entity contains clinical data collected during a subject’s visit. We chose the EpisodeOfCare resource for this entity because it provides details about the group of activities and their purpose pertaining directly to a patient. A study event may result in many visits from a patient. Each individual visit is modelled as an Encounter and is linked to the episode of care through the episodeOfCare attribute. The patient attribute links the resource to the study subject while the assessor attribute provides a link to the clinician conducting the clinical assessment.

### Form

A form defines a collection of data items collected during the study and termed a *case report form*. A form comprises a FormDef and a FormData component that are referenced using a common OID. The form is linked to CarePlan through the activity. actionResulting attribute. The FormDef defines the form structure and its questions. The logical mapping of forms in FHIR is the Questionnaire resource. This resource contains the typical attributes for questionnaires, such as an identifier, version, publisher and status, but can also be customised using the extension mechanism in FHIR. The FormData entity contains the clinical data associated with the form. The logical mapping for the FormData in FHIR is the QuestionnaireResponse resource. The benefits of using the QuestionnaireResponse resource are that the order of the responses is maintained and these can be linked and validated against the questions asked. Conversely, however, few mechanisms exist to standardise the generation of CRFs for clinical studies [8, 11]. This limits the reuse of CRFs *unchanged* across protocols [11]. Furthermore, the tendency is to organise data items, relevant to a research protocol, into individual CRFs based on considerations other than logical grouping [8, 11] but one that befits the data capture process [8]. Owing to the strong coupling between the form design and the ODM model, until such a time that implementations of ODM allow for a clear demarcation between the form design and its display, we advise against modelling the CRF *per se* as a FHIR resource [7].

### Item group

The ItemGroupDef and ItemGroupData entities constitute an item group referenced using a common OID. The ItemGroupDef entity defines the optional grouping of questions on a form. Groups are defined using the Questionnaire.group attribute. The FHIR specification stipulates that a group attribute define either a question or a group but not both. The ItemGroupData contains the clinical data detailing the responses for the item group. FHIR organises these grouped responses within the QuestionnaireResponse.group attribute. Similar to forms, items are often grouped to match the data collection process and not necessarily because of their semantic similarity [8, 11].

### Item

At the item level, the ItemDef and ItemData entities define each question and its subsequent response. The ItemDef entity defines the question asked during the study along with defining attributes such as the datatype, data size, measurement unit, permissible range and code list. The Questionnaire.group.question attribute is the most appropriate to define the ItemDef entity. The logical mapping for the ItemData entity is the QuestionnaireResponse.group.question attribute. The response to the question is then contained within the question.answer sub-attribute. This model works best in a lifestyle study scenario using questionnaires in the traditional *question-answer* mode. In the case of longitudinal clinical studies where the responses are analogous to a patient’s observations during an episode of care, we believe the ItemData entity to be more appropriately represented using the Observation resource. Furthermore, as outlined in the FHIR specifications, data captured in questionnaires can be difficult to query after the fact. Individual items within a QuestionnaireResponse or an Observation are subsequently linked back to the Encounter in which they occur.

### Discussion

An implementation of the mapping between ODM and FHIR is available at http://healthinet.it.csiro.au/net/jbs/odmFhir. We have semantically enriched the original ODM data with relevant domain information from SNOMED CT^4^ and LOINC^5^. The implementation demonstrates that the FHIR resources provide a good fit to semantically enrich the extracted data from the CDISC ODM. In spite of its shortcomings in providing context to the clinical data, the CDISC ODM provides a sound hierarchical framework for capturing the clinical data. However, as outlined in [25], a mapping process invariably leads to the loss of pertinent information. On the metadata side, for example, a study is modelled as a CarePlan. The CarePlan resource, however, is not used in its intended manner in that it does not relate to a particular individual. Similarly, despite being chosen to capture the clinical data, the ClinicalImpression resource has no capability to model the study hierarchy. As a result, it relies on several other FHIR resources, such as EpisodeOfCare and Encounter, which are also not used as intended, to describe the hierarchy. As stated by Kubick [24], it is preferable to avoid data transformations, if possible, especially when this involves *massaging* the data to fit into different formats, as this opens up the possibility of introducing errors and reducing the data reliability. Another issue relates to discrepancies between the data types defined within the ODM and FHIR models. In addition to the type, ODM allows the permissible range of the resulting data and, in the case of decimal values, the length of the permissible value to be defined. The answer attribute within the QuestionnaireResponse resource has no such capability. The Observation resource is the only one to allow such a definition.

The main challenge of the mapping process, however, relates to the FHIR specifications. Being an emerging and evolving standard, FHIR is in a great state of flux. As such, FHIR resources are constantly being updated between releases. The implications are that relationships described using one version of the FHIR specifications may no longer be available in a subsequent version. The Questionnaire and ClinicalImpression resources are two resources that have undergone several changes.

## Clinical study design using FHIR

**Fig. 4:**
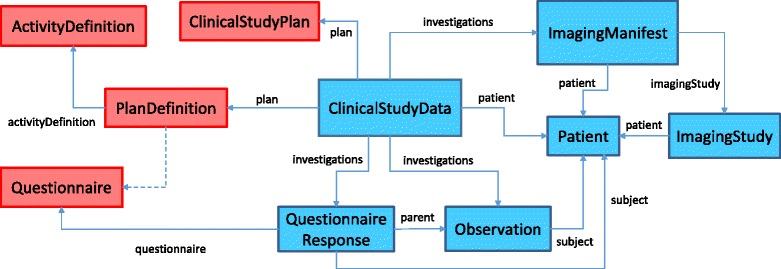
The clinical research data model in FHIR. Illustrates the *metadata* (red) and *data* (blue) resources comprising the clinical data model for describing and capturing the research study *natively* in FHIR. The study plan can be described using either the ClinicalStudyPlan or PlanDefinition resource. The latter can be further defined using the ActivityDefinition resource. The Questionnaire resource provides the definition for forms within the study plan. A link to the study plan is contained within the ClinicalStudyData resource. The ClinicalStudyData resource encapsulates the clinical data comprising the research study. It facilitates links to the Patient resource, to describe the study participant. It further describes *investigations* that can be a QuestionnaireResponse or a series of Observation or ImagingManifest resources. The ImagingManifest resource further defines an ImagingStudy resource to describe the imaging study being conducted

**Fig. 5:**
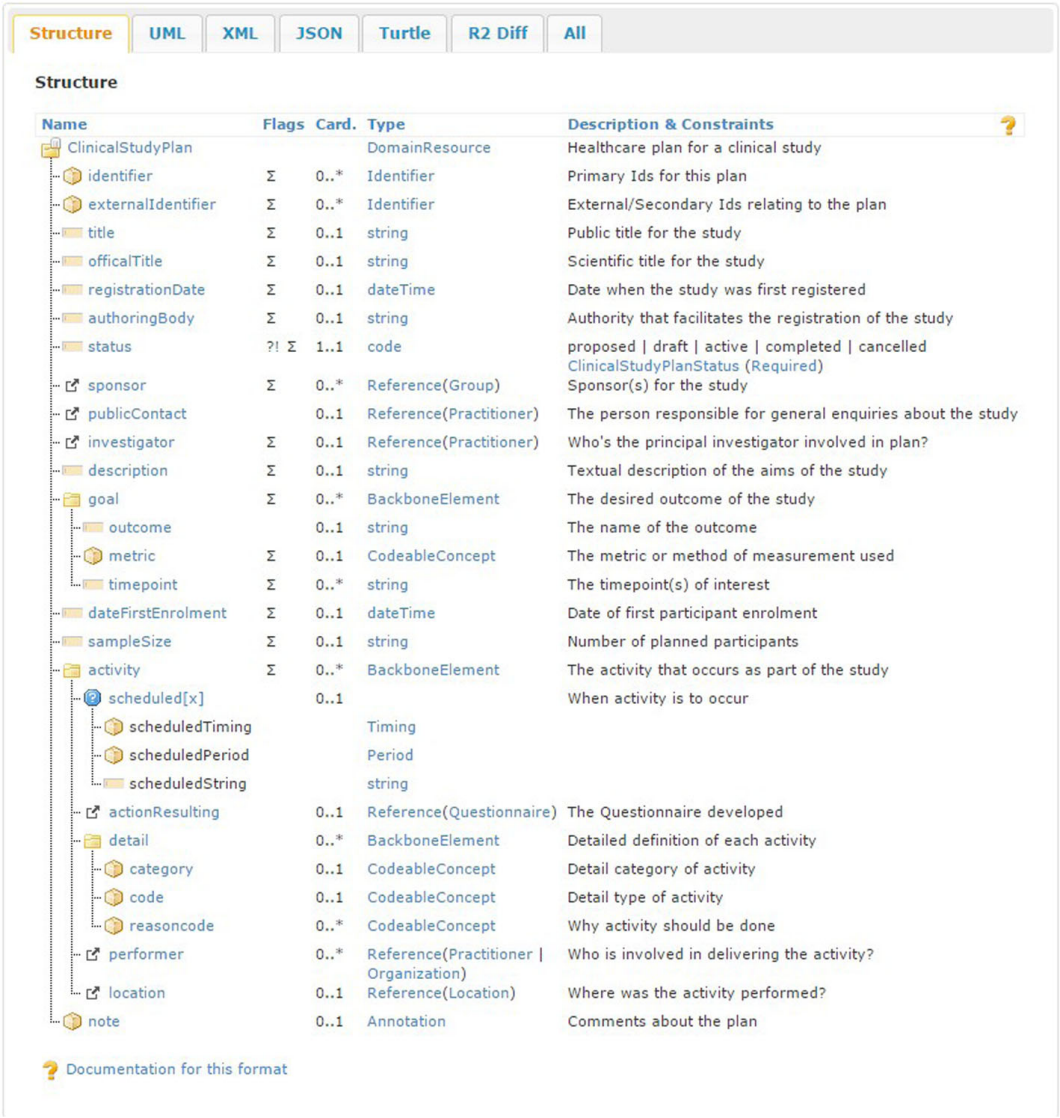
The ClinicalStudyPlan resource. Describes the elements comprising the ClinicalStudyPlan resource. This resource has been generated using the FHIR Build Process [45] based on the FHIR Guide to Designing Resources [46]. The build process builds the resource and generates the webpage that describes the resource, as depicted in this Figure. The table structure is defined in the Resource Definition page [47], which also provides a definition of the flags; ‘?!’ indicates that the element is a modifying element, while ‘*Σ*’ indicates that this element is part of the summary set. The activity element allows either the definition of detailed items or a Questionnaire resource to be specified

**Fig. 6:**
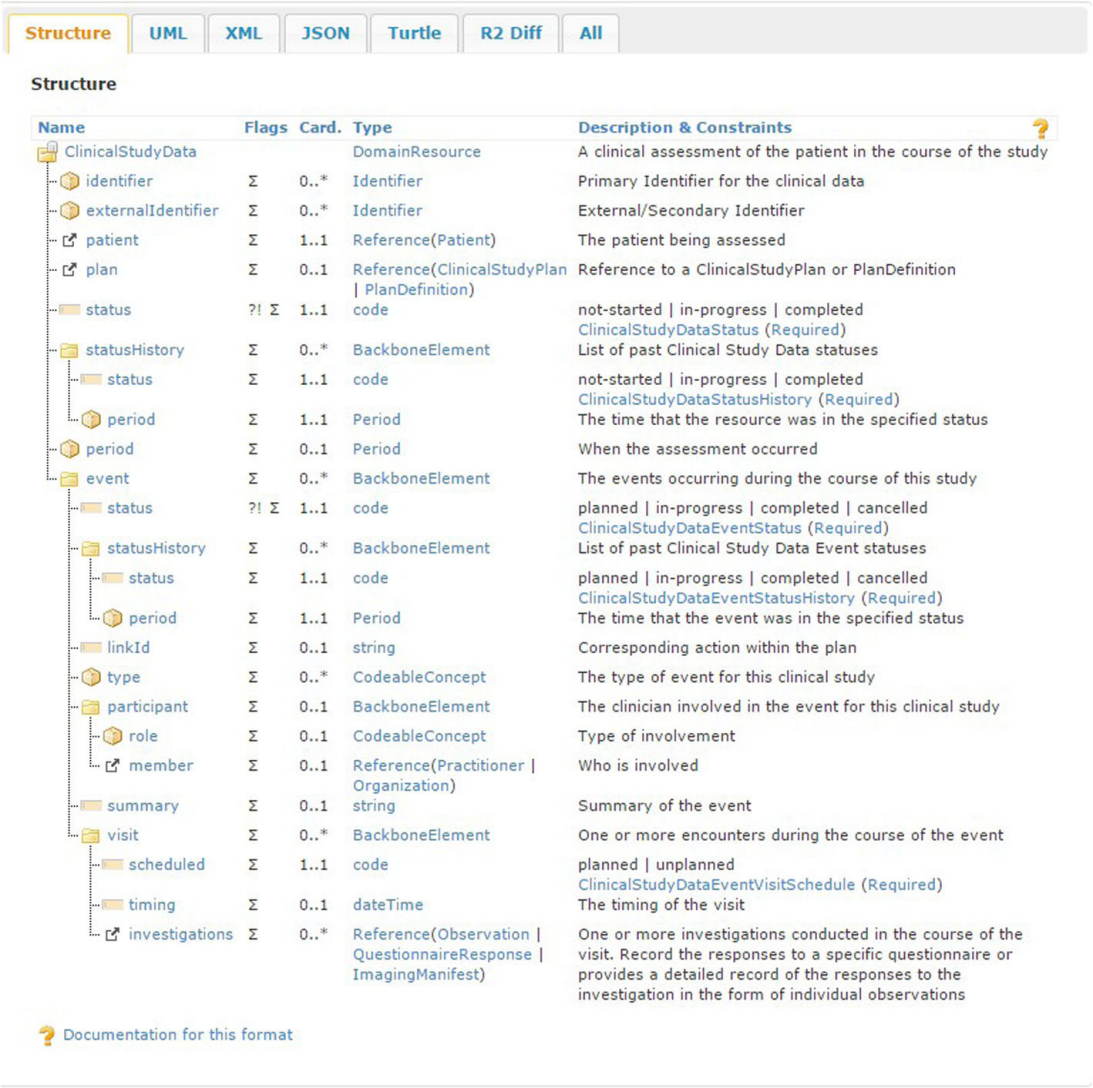
The ClinicalStudyData resource. Describes the elements comprising the ClinicalStudyData resource. This resource has also been generated using the FHIR Build Process [45] based on the FHIR Guide to Designing Resources [46]. The table structure is defined in the Resource Definition page [47], which also provides a definition of the flags; ‘?!’ indicates that the element is a modifying element, while ‘*Σ*’ indicates that this element is part of the summary set. The event element describes the events occurring throughout the study. An event can be further divided into visits. Each visit defines an investigation, which can be only one of the following: a QuestionnaireResponse resource or a series of Observation or ImagingManifest resources

Kubick [24] advocates (i) for the adoption of FHIR for clinical research; (ii) for clinical data to be captured directly at the source; and, (iii) for data transformation to be avoided whenever possible. Similarly, Huser et al. [10] argue that the adoption of a single format for study protocols and study results decreases the development time required to import studies into the repository or to exchange data between systems. Besides, the FHIR model has the potential to manage clinical data in its own right [8]. Consequently, we propose the introduction of two new FHIR resources to capture the data and metadata from the clinical study. These resources have been integrated in a data model, as illustrated in Fig. 4, which corresponds to the mapping, in FHIR, for a typical research study. The ClinicalStudyPlan resource, outlined in Fig. 5, defines the study and provides an overview of the planned activities. The ClinicalStudyData resource, outlined in Fig. 6, describes the data captured as part of the study organised around the events and visits of the patient.

### ClinicalStudyPlan

The ClinicalStudyPlan resource comprises several attributes to capture the fundamental concepts within the study. Thus the identifier attribute provides a unique identifer for the resource. A title attribute captures the title under which the study is publicly known. An officialTitle attribute holds the scientific title of the study. The date of registration of the study is contained in the registrationDate field and the regulatory agency effecting the registration is depicted within the authoringBody field. A mandatory status attribute specifies the current state of the resource. The study sponsors can be described in the sponsor field, which allows a Group resource to be defined. The publicContact attribute specifies the contact details of the person responsible for general enquiries about the study. An investigator attribute discloses the principal investigator for the study; a person tasked at initiating the study, developing the study protocol and responding to scientific enquiries about the study. A textual description of the aims of the study is provided by the description attribute. The actual or forecasted date of first participant enrolment is recorded in the dateFirstEnrolment and the expected total number of participants enrolled is captured in the sampleSize attribute.

The desired outcome of the study is captured within the goal attribute. Each goal is further divided into three sub-attributes. The name of the outcome is contained within the outcome attribute. A metric attribute describes the metric or method of measurement used to evaluate the outcome and finally a timepoint attribute records the timepoints of interest in which to achieve the goal.

We then define the activities that constitute the study. In [8], we outlined how the Questionnaire resource is insufficient to capture all activities from clinical studies, especially longitudinal ones. By defining all aspects of actions resulting from clinical studies within activity attributes, we facilitate the definition of both traditional questions and more observational measurements. A scheduled attribute allows the timing of an activity to be defined. We chose an actionResulting attribute to describe the questionnaire developed as part of the activity. We then define a detail attribute to provide a detailed description of sub-activities that will ultimately lead to Observation and ImagingManifest resources in FHIR. This attribute thus provides three sub-attributes to document the category, type and rationale for each sub-activity. We also chose to record the Practitioner or Organisation involved in the activity through the performer attribute and provide a reference to the activity’s location using a location attribute. Finally, a note attribute allows any comments relating to the clinical study plan to be recorded.

We have started engaging with the HL7 FHIR-I^6^ [26] and RCRIM^7^ [27] working groups. The FHIR community, however, intends to release, as part of STU3^8^, a PlanDefinition resource that captures many of the functionalities of the ClinicalStudyPlan resource.

### PlanDefinition

PlanDefinition ^9^ is a resource proposed by the HL7 community that is at the *ballot* phase and that they intend to release as part of STU3 in late 2016. We will only comment on the main concepts as this is a draft proposal that is still subject to change at short notice. Unlike the ClinicalStudyPlan, this resource has not been designed to address the planning of clinical research specifically but it is flexible enough to undertake this role. However, similar to the ClinicalStudyPlan resource, it contains attributes to represent the plan’s unique identifier, name, status, purpose and contributor. In addition, it defines the version as well as attributes that capture the type of plan defined, the clinical usage foreseen for the plan, a natural language description of the plan, dates of publication and last review, the context of use (coverage) of the plan as well as the topics described.

Central to this resource is the definition of actions (actionDefinition) to occur as part of the plan. Each action has an identifier, a label, a title, a description of the action both in natural language and as Codeable entities and a link to supporting documentation for the action. Each action further defines a condition for whether as well as some triggers to specify when the action should occur. A description of the activity comprising this action can be further defined within an ActivityDefinition resource.

The proposed ActivityDefinition ^10^ resource provides a *conceptual* description of an action that should be undertaken. Similar to the PlanDefinition resource, it is at the *ballot* phase and is intended to be released as part of STU3 in late 2016. It contains similar organisational attributes as the PlanDefinition resource. A detailed definition of the activity can be achieved using a CodeableConcept element. A category attribute defines the type of activity undertaken and a timing attribute specifies when the activity should occur.

### ClinicalStudyData

The ClinicalStudyData resource describes the data captured during the study. An identifier is defined to provide a link to the primary identifiers for the study. For external identifiers, such as a hospital patient id, an externalIdentifier attribute is provided. A mandatory patient attribute provides a reference to the patient being assessed. The ClinicalStudyData resource provides a link to either the ClinicalStudyPlan or PlanDefinition resource, through the plan attribute, to uniquely identify the study that this clinical data instance represents. A status attribute defines the current state of the resource. We also chose to keep a record of past statuses in a statusHistory attribute that captures the past statuses as well as the time that the event was in the specified state. The time period during which the patient underwent the clinical assessment is depicted in the period attribute.

We then define the event occurring during the course of the study. An event represents the execution of one or more activities during the course of the study to assess the patient. Each event transitions through a number of states and the state is contained within the status attribute. A type attribute describes the type of the event. The clinicians involved in this event are described within the participant attribute, which further defines their role and a reference to the involved member, be it a Practitioner or an Organization. A summary of the event is provided within the summary attribute.

In clinical study parlance, an event can last anything from a few seconds to several months or even years. Consequently, we define a visit attribute to describe one or more encounters between the clinician and the patient. Visits can be *planned* or *unplanned* and this is defined within the scheduled attribute. The timing of the visit is described within the timing attribute. Finally, we define an investigations attribute to capture one or more clinical investigations during the course of the visit. These take the form of a reference to either a QuestionnaireResponse or a series of Observation or ImagingManifest resources.

## Demonstrating the clinical study design with FHIR

We illustrate the fit of the FHIR data model by discussing a clinical study focussed on cardiovascular episodes. Our focus is to highlight the impact that the addition of contextual information, and their relationships with the data elements, have on the semantic relevance and interpretation of the clinical data. Typically, the output from a clinical study, in ODM XML format, is as depicted below:



This states that the study participant has a *blood pressure* of 119/79 mmHg but provides no information on how the measurements were obtained. Handler [28] outlines nine factors that may affect the accuracy of blood pressure measurements. To assist the user in making informed decisions about the clinical data, relevant contextual information, such as illustrated below, should be provided with the data. This additional metadata tells us that the readings were taken at *10:00 am* by a *nurse* from the *left upper arm* and in a *sitting* position. While it is important to standardise the data and metadata, what is missing is the relationship to the initial blood pressure measurements. When the measurements are presented as a series of unrelated data elements, they cannot reliably be interpreted (Appendix in [5]).



The FHIR framework, in particular resources such as Observation, provides the means to accurately represent the relationships between the data elements so that they can be understood and interpreted more effectively. A representation of the blood pressure above, implemented using the Observation resource, is illustrated in the Additional file 1. In addition to capturing the blood pressure measurements described previously, the Observation resource provides a reference to the Patient resource to identify the study participant and to the Practitioner resource to identify the clinician performing the blood pressure measurement. More importantly, it natively encapsulates the contextual information, such as the *body part* and *body position*, as well as the ability to interpret the measurements.

By taking advantage of resources that encapsulates rich interrelated clinical data, as demonstrated by the Observation resource, the ClinicalStudyData resource facilitates the definition of the entire research study, in terms of the subjects enrolled; the clinical data associated with these subjects and the experiments undertaken. In addition, it provides a framework, through the plan element, for the study plan to be associated with the clinical data. An implementation of the ClinicalStudyData and ClinicalStudyPlan resources is provided at http://healthinet.it.csiro.au/net/jbs/.

## Discussion

The implementations of the ClinicalStudyPlan and ClinicalStudyData resources demonstrate the fit of the FHIR standard in capturing and managing clinical data from research studies. The pertinence of this finding is that the clinical data no longer need to be transformed from an arbitrary standard into FHIR resources, thus reducing the risk of introducing errors and losing fidelity. The proposed models achieve semantic interoperability by defining a set of common elements for describing the actions performed on the data as well as defining common elements for describing the data and its context through the use of controlled terminologies and ontologies. This, then allows the resources to be shared and processed across systems. The FHIR resources provide the means to navigate and access the clinical data at numerous levels with the addition of several dimensions at the *patient*, *event*, *activity* and *data item* level, thereby negating the limitations of the monolithic and rigid hierarchy of the ODM data model.

The World Health Organisation (WHO) has released a list of twenty mandatory items for the definition of a study protocol [29] so that the given trial can be considered fully registered. We present, in Table 1, a listing of the twenty items alongside the attributes from the ClinicalStudyPlan and PlanDefinition resources. We have not provided a study type as the ClinicalStudyPlan inherently suggests a clinical study. We have also chosen not to explicitly define the source of monetary funds and countries of recruitment as these are primarily associated with *clinical trials*. However, it is our support for eligibility criteria that is particularly inadequate. In our defence, our focus here has been the definition of an alternative *structural* representation to CDISC ODM for clinical study design. Furthermore, we regard the formulation of an effectual eligibility criteria as non-trivial and one that we deemed out of scope for this paper. We intend to engage with the HL7 community to embed computable study protocol criteria within our resource as adequate representation of the study protocol is very useful and important [10]. Previous attempts, such as the CDISC Protocol Representational Model (PRM), have had limited adoption by the clinical study community [10]. (PRM [30] is a UML^11^-based standard that developed a set of standardised protocol concepts that was intended to be used alongside the other CDISC and HL7 standards.)

**Table 1:** Listing of the 20 WHO items for clinical study protocol

WHO trial registration data set	PlanDefinition	ClinicalStudyPlan
1. Primary registry and trial identifying number	Identifier	Identifier
2. Date of registration in primary registry		RegistrationDate
3. Secondary identifying numbers	Identifier	ExternalIdentifier
4. Source(s) of monetary or material support		
5. Primary sponsor		Sponsor
6. Secondary sponsor(s)		Sponsor
7. Contact for public queries	Publisher	PublicContact
8. Contact for scientific queries		Investigator
9. Public title	Title	Title
10. Scientific title		OfficialTitle
11. Countries of recruitment		
12. Health condition(s) or problem(s) studied	Purpose^*^	Description
13. Intervention(s)	ActionDefinition	Activity
14. Key inclusion and exclusion criteria		
15. Study type	Type^*^	*
16. Date of first enrolment		DateFirstEnrolment
17. Target sample size		SampleSize
18. Recruitment status	Status	Status
19. Primary outcome(s)	Coverage^*^	Goal
20. Key secondary outcomes	Coverage^*^	Goal

An asterisk (‘*’) indicates that this data item is partially or indirectly addressed in the model

The appeal in definining a visit as part of an event in the ClinicalStudyData resource is to more accurately describe protracted events within multimodal longitudinal clinical studies. It is often useful, in the case of lengthy events, to be able to define a *sub-event* and subsequently record the study participants’ attendance to the sub-event. Consequently, the outcome of those visits can be represented as a QuestionnaireResponse, ImagingManifest or an Observation through the investigations attribute. The QuestionnaireResponse, ImagingManifest and Observation resources suit different types of clinical studies [8]. The pertinence of the Observation resource is the ability to store important contextual information alongside the clinical data, the ability to interpret the observation in the context of a controlled vocabulary or ontology and the ability to provide some justification as to the absence of a measurement [8].

While the PlanDefinition resource can be used to describe the study plan, it still has some inadequacies to overcome. As the PlanDefinition resource has not been specifically designed to address the planning of clinical research, it logically has to be more generic. Consequently, it is unclear how the PlanDefinition resource relates to the FHIR resources designed to capture the clinical data that it defines. Furthermore, it is also unclear what mechanism is envisaged to ensure that the data capture resources conform to the plan definition. Moreover, as the resource has not been designed for a clinical research domain, the PlanDefinition resource also lacks the necessary mechanisms to fully define the study protocol. In particular, it does not offer the option of recording the date of registration, the sponsor(s), the date of enrolment, expected sample size, study type and study outcome(s). More importantly, in our view, is the lack of support for *machine-processable* inclusion and exclusion criteria to be embedded within the PlanDefinition resource. While the PlanDefinition defines a trigger and a condition element, these relate to the execution of the PlanDefinition resource and do not constitute the definition of the conditions addressing the eligibility of the participants to participate in the study. We advocate for the eligibility criteria to be designed in a manner to influence and advance the study design and form generation as outlined in [3] in their five phases of clinical research data lifecycle.

While the ClinicalStudyPlan and PlanDefinition resources are *structurally similar*, there are subtle differences between them. It is unclear how the Questionnaire resource (indicated by dotted lines in Fig. 4) fits within the PlanDefinition resource. This may, in our view, restrict its ability to be used for anthropological studies or surveys.

### Related work

Prior to FHIR, several information models have been proposed to standardise the representation of clinical information. The Clinical Element Model (CEM) is an information model designed to provide a consistent architecture for representing clinical information in EHR systems [31]. The ISO 13606 standard is an international standard published by ISO that specifies the information models and vocabularies needed for the interoperability of EHR systems [32]. Both models aim to address the issue of semantic interoperability by standardising the data, metadata and their relationships similar to our approach.

Numerous research have centred around the CDISC models recently. Dugas [25] describes two tools to convert forms between the CDISC ODM and HL7 CDA^12^ formats to facilitate the sharing of electronic health records (EHRs) and clinical data to address the problem of redundant documentation in both systems. His findings reflect our position that the conversion process is *lossy* because the CDISC and HL7 models serve different purposes and hence have different properties. Similarly, the SALUS project [33, 34] is a former attempt to adapt CDISC standards to build a semantic framework to improve the interoperability between clinical research and clinical care domains. More specifically, it looks at combining the strengths of CRFs with those of EHRs to address adverse drug reactions. We envisage our proposed FHIR clinical study model to facilitate the incorporation of existing EHR data to augment the capabilities of retrospective observational studies similar to their approach. Jiang et al. [35] have developed and evaluated a Semantic Web-based approach for the generation of domain-specific templates from the integration of the BRIDG model and the ISO 21090 data types, to support clinical study metadata standards development. Vadakin and Hinkson [36] discuss the CDISC PRM and outline its importance in supporting research study design, registration, tracking and in providing a single-source of protocol content electronically. They stress that typical protocol document is not useful for information management and re-use. PRM standardises the protocol content into a structured document that is easier to understand and to exchange, in machine-readable format, across systems [36]. We are mindful of their findings in order to address the issue of the protocol definition within our research data model.

A topical area of research has been the standardisation and structuring of clinical forms. Abler et al. [18] discuss the need for a language for forms that can effectively record the logical relationships between questions or sets of questions asked in the forms. Richesson and Nadkarni [11] provide a review of the electronic data capture standards landscape and discuss their current limitations. Bruland et al. [9] discuss the standardisation of CRFs to achieve interoperability in clinical research. They outline the difficulties of promoting the standardising and structured representation of forms in the context of data exchange and propose a mapping model between the National Cancer Institute forms and CDISC ODM files semantically annotated using the *Alias* element. As stated in [8], the tendency would be to organise the forms within a Questionnaire resource in FHIR. However, this understates the nature of the information captured and the choice of a QuestionnaireResource, Observation and ImagingManifest resource ensures the optimal capture of the information.

The Linked Clinical Data Cube (LCDC) [6, 7, 19] describes a *semantic web* approach to investigate the association of the semantic statistics vocabularies with clinical data exchange standards and demonstrate their fit in achieving the semantic enrichment of clinical study data with a view to fulfilling semantic interoperability. The LCDC defines a set of modularised data cubes that helps manage the multi-dimensional and multi-disciplinary nature of clinical data. It requires mapping to the RDF Data Cube [37] and DDI^13^-RDF Discovery [38] vocabularies to organise the data and links to domain ontologies to semantically enrich it. The LCDC represents the precursor to our data model in FHIR. The HL7 working group on Semantic Interoperability [39] has initiated work on translating the XML and JSON version of FHIR into FHIR RDF. Once completed, this should allow the integration of the FHIR data model with the semantic statistics vocabularies.

### Future work

We intend to engage with the FHIR community to address the full support for the definition of eligibility criteria within the FHIR resources. There is a need for the current text-based criteria to be formalised and provided in machine-readable format to facilitate computerised determination of eligibility [10]. Machine-processable definition of eligibility criteria will not only mould the study design but can influence the patients’ recruitment process as outlined in [40].

The FHIR specification provides the functionality, through the FHIR mapping language [41], to transform clinical data from one model to another. We intend to take advantage of this functionality to map the FHIR clinical data model back to the CDISC standards. As outlined earlier, the regulatory bodies favour the use of the CDISC standards for the reporting of clinical studies [5]. By using FHIR to model the clinical study data, we capture the contextual information, and fulfil the requirements of the FDA by retrofitting the clinical data to the CDISC models.

We also aim to support the formulation of temporal constraints to assist in the scheduling of activities as outlined in [42], which describes a knowledge-based approach to specifying and monitoring temporal constraints in relational databases.

## Conclusion

This paper has presented a proposal for a clinical study architecture to support the semantic interoperability of clinical data using the FHIR resources. We have shown how the clinical research community is likely to benefit from the adoption of FHIR resources to capture and manage clinical study data. In this regard, we have outlined a method to link clinical data from the XML-based CDISC ODM model to a selective group of FHIR resources. While we have revealed a fit between the ODM model and the FHIR resources, we do not regard this as a long term solution. First, owing to the evolving nature of the FHIR specifications, this mapping is likely to change at a whim. Second, it is preferable to avoid data transformations but for data to be captured directly at the source. We have thus proposed two FHIR models, a ClinicalStudyPlan and a ClinicalStudyData resource, and shown that they can natively manage clinical data. We have compared our work to the proposed HL7 PlanDefinition FHIR resource and discussed their suitability in adequately representing the research study protocol definition. We have demonstrated, with the help of a working example, the fit of our clinical data model in interpreting clinical research data. Our work has built the foundations to not only facilitating the *syntactic* but also *semantic* interoperability of clinical research data.

## Endnotes

^1^ Extensible Markup Language [43]

^2^ Health Level Seven

^3^ Based on the ‘Standard for Trial Use 3’ September 2016 version

^4^ Systematized Nomenclature of Medicine Clinical Terminology

^5^ Logical Observation Identifiers Names and Codes

^6^ FHIR-Infrastructure

^7^ Regulated Clinical Research Information Management

^8^ Standard for Trial Use 3

^9^ This resource can be found at http://hl7.org/fhir/2016Sep/plandefinition.html

^10^ This resource can be found at http://hl7.org/fhir/2016Sep/activitydefinition.html

^11^ Unified Modeling Language

^12^ Clinical Document Architecture

^13^ Data Documentation Initiative

## Additional file

Additional file 1 The file observation_example.json lists the Observation resource as described in the *Demonstrating the clinical study design with FHIR* section in json format. (JSON 2 kb)

## Abbreviations

CDA: Clinical document architecture
CDASH: Clinical data acquisition standards harmonization
CDISC: Clinical data interchange standards consortium
CEM: Clinical element model
DSTU2: Draft standard for trial use 2
FHIR: Fast Healthcare interoperable resources
FHIR-I: FHIR-Infrastructure
HL7: Health level seven
ISO: International organization for standardization
LCDC: Linked clinical data cube
LOINC: Logical observation identifiers names and codes
ODM: Operational data model
RCRIM: Regulated clinical research information management
SNOMED CT: Systematized nomenclature of medicine clinical terminology
STU3: Standard for trial use 3
XML: Extensible markup language
